# Population attributable risk for diabetes associated with excess weight in Tehranian adults: a population-based cohort study

**DOI:** 10.1186/1471-2458-7-328

**Published:** 2007-11-14

**Authors:** Farhad Hosseinpanah, Mehdi Rambod, Fereidoun Azizi

**Affiliations:** 1 Obesity Research Center, Research Institute for Endocrine Sciences, Shaheed Beheshti University of Medical Sciences, Tehran, Iran; 2 Endocrine Research Center, Research Institute for Endocrine Sciences, Shaheed Beheshti University of Medical Sciences, Tehran, Iran

## Abstract

**Background:**

Little evidence exists regarding the magnitude of contribution of excess weight to diabetes in the Middle East countries. This study aimed at quantification of the impact of overweight and obesity on the incidence of type 2 diabetes mellitus (T2DM) at a population level in Tehran, Iran.

**Methods:**

Using data of a population-based short-term cohort study in Iran, which began in 1997 with 3.6-year follow-up, we calculated the adjusted odds ratios (OR) and population attributable risks (PAR) of developing T2DM, i.e. the proportion of diabetes that could have been avoided had overweight and/or obesity not been present in the population.

**Results:**

Of the 4728 subjects studied, aged ≥ 20 years, during the 3.6-year follow-up period, 3.8% (n = 182) developed T2DM. This proportion was 1.4%, 3.6%, and 7.8% for the normal, overweight, and obese subjects, respectively. When compared to normal BMI, the adjusted ORs for incident diabetes were 1.76 [95% confidence interval (CI) 1.07 to 2.89] for overweight and 3.54 (95% CI 2.16 to 5.79) for obesity. The PARs adjusted for family history of diabetes, age, triglycerides, systolic blood pressure was 23.3% for overweight and 37.1% for obesity. These figures were 7.8% and 26.6% for men and 35.3% and 48.3% for women, respectively.

**Conclusion:**

Incident T2DM is mainly attributable to excess weight, significantly more so in Tehranian women than men. Nonetheless, the contribution of excess weight in developing T2DM was lower in our short-term study than that reported in long-term periods. This probably reflects the significant role of other risk factors of T2DM in a short-term follow-up. Hence, prevention of excess weight probably should be considered as a major strategy for reducing incidence of T2DM; the contribution of other risk factors in developing T2DM in short-term period deserve to be studied and be taken into account.

## Background

Progressive increment of body weight of the population is a major problem faced by the public health systems in a number of countries. Today, more than 1.1 billion adults worldwide are overweight, of which 312 million are obese [1]. In the past 20 years, the rates of obesity have tripled in developing countries adopting Western lifestyles. In Iran, as well as other developing countries, the prevalence of overweight and obesity shows a rapid increasing pattern during recent years [2].

Obesity and overweight are associated with a number of co-morbidities [3]. A higher body-mass index (BMI) has been shown to account for up to 16% of the global burden of disease, expressed as a percentage of disability-adjusted life-years. In the developed world, 2 to 7% of total health care costs are attributable to obesity. Moreover, the growing prevalence of type 2 diabetes, cardiovascular disease, and some cancers are tied to excess weight. Several studies have found that the single measure of being overweight or obese in early middle age predicts increased cardiovascular disease risk two or three decades later [4-7] and that the increase in the prevalence of type 2 diabetes is closely linked to the upsurge in obesity [8-11]. Based on the fact that adipose tissue is an important endocrine organ, it is not surprising that overweight individuals are at increased risk of diabetes [12,13]. Indeed, obesity is the strongest risk factor for diabetes [14]. A 20-year follow-up of Whickham study found that BMI is an independent predictor of diabetes [15]. Another study from Japan revealed that increases in BMI of 1 kg/m^2 ^(equal to body-weight gain of 2.4 to 2.9 kg) may raise the risk of diabetes by 25% [14]. BMI, even within the non-obese level, is still a dose dependent risk factor for diabetes mellitus [14,16]. A recent survey of a Scottish population reported that within the normal weight group, the risk of developing diabetes was 2 to 3 times greater in the higher half of normal BMI range in comparison to the lower half [17].

Considering the fact that little evidence exists regarding the contribution of excess weight to diabetes in the Middle East countries, in this cohort study with 3.6-years follow-up, we examined the strength of association between excess weight and incident type 2 DM in a short term period, using data of a large population-based cohort study conducted in Iran [18]. Moreover, we aimed at quantifying the adjusted population attributable risk percentage of developing type 2 DM, an estimate that can give us an idea of the extent of diabetes mellitus caused by overweight and obesity.

## Methods

### Subjects and Design

The Tehran Lipid and Glucose Study (TLGS) is a study being conducted to determine the prevalence of non-communicable diseases and the risk factors of atherosclerosis among Tehran's urban population and to develop population-based measures and lifestyle modifications to decrease the prevalence and prevent the rising trend of diabetes mellitus and dyslipidemia [18,19]. The design of this study includes two major components, a cross-sectional prevalence study of cardiovascular disease and associated risk factors and then, a prospective 20-year follow-up in several phases, at approximately 3.6-year intervals. A multi-stage stratified cluster random sampling technique was used to select 15005 people, aged 3 years and over, from urban district 13 of Tehran, the capital of the Iran; the district is located in the center of Tehran and the age distribution of its population is representative of the overall population of Tehran. During sampling, the list of all households under the coverage of the district's three healthcare centers (the official bodies responsible for vaccination programs and collection of health-related statistics in a district) was used. Then, a random sample of households, stratified according to healthcare centers to achieve a distribution similar to the original population, was chosen, and from each household, all members above the age of three were recruited. The study began in December 1997 and the cross-sectional phase completed in 2000; the first follow-up survey began in 2001 and completed in 2004. After excluding subjects aged < 20 years (n = 4642), those with known or newly diagnosed type 2 diabetes (n = 1164), those with missed values of weight, height, or other variables (n = 349), or lost to follow-up (n = 4122), data of 4728 subjects with a complete 3.6-year follow-up was used in this cohort study. In comparison to those completed the follow-up, individuals lost to follow-up had lower value of systolic blood pressure (119 vs. 117 mmHg), BMI (26.9 vs. 26.2 Kg/m^2^), waist circumference (88.2 vs. 86.5 cm), triglycerides (1.88 vs. 1.78 mmol/L), fasting plasma glucose (4.99 vs. 4.94 mmol/L), and 2-h postchallenge plasma glucose (5.94 vs. 5.83 mmol/L).

At the beginning of the cross-sectional phase, all participants provided written informed consent, which was approved by the institutional ethics committees (Research Institute for Endocrine Sciences) and was conducted in accordance with the principles of the Declaration of Helsinki. Thereafter, collection of demographic data and anthropometric examinations were undertaken by trained general physicians. Weight and height were measured with subjects wearing normal indoor clothing, but not shoes. Weight was recorded using a Seca 707 weighing machine (range: 0.1–150 kg) with an accuracy of up to 100 gr. The machine was regularly checked for precision after every 10 measurements. Height was measured without shoes using a tape stadiometer with a minimum measurement of 1 mm. Body mass index (BMI) was calculated by dividing weight (in kilograms) by height squared (in meters). To measure systolic blood pressure (SBP) and diastolic blood pressure (DBP), participants were initially made to rest for 15 min; then a qualified physician measured blood pressure twice in a seated position using a standard mercury sphygmomanometer on the right arm and, the mean of the two measurements was considered as the participant's blood pressure. Fasting plasma glucose (FPG), 2-hour postchallenge plasma glucose (2h-PG) and serum lipids were measured at the TLGS research laboratory on the day of blood collection. Details of the biochemical measurements are published elsewhere [20].

### Definitions

BMI was first categorized according to the World Health Organization (WHO) definitions of underweight (< 18.5 kg/m^2^), normal (18.5 to < 25 kg/m^2^), overweight (25 to < 30 kg/m^2^), and obese (≥ 30 kg/m^2^) [21]; excluding the underweight subjects, the other categories BMI were then divided into two subcategories (18.5 to < 22.5 kg/m^2 ^and 22.5 to < 25 kg/m^2^; 25 to < 27.5 kg/m^2 ^and 27.5 to < 30 kg/m^2^; 30 to < 32.5 kg/m^2 ^and ≥ 32.5 kg/m^2^). Family history of diabetes was considered as positive if any of the first-degree relatives of the subject had diabetes. For the current study, diabetes was defined as FPG ≥ 7.0 mmol/L and/or 2h-PG ≥ 11.1 mmol/L or current use of oral anti-hyperglycemic medications. Smoking statuses were defined according to WHO guidelines [22]; current smoker was defined as a person who smokes cigarettes daily or occasionally; ex-smoker was defined as a person who was formerly a daily or occasional smoker, but currently did not smoke at all and a non-smoker was defined as a person who has never smoked before or has smoked very little in the past.

### Statistical Analyses

Continuous data are expressed as mean ± SD and categorical data are expressed as percentage. Continuous and categorical variables were compared using the student *t*-test and the χ^2 ^test, respectively. Differences between more than two groups were analyzed using ANOVA with the Tukey post hoc test. Trends of proportions were examined using linear-by-linear χ^2 ^test and trends of continuous data were examined using simple linear regression models. Logistic regression models were used to assess the unadjusted and adjusted odds ratios (OR) of incident diabetes across categories of BMI [normal (as reference category), overweight, and obese] in all the population and then in each sex, separately. Family history of diabetes, age (at the cross-sectional phase of the study), triglycerides, and systolic blood pressure were entered in stepwise manner to obtain adjusted ORs of incident diabetes. While some of these variables, such as hypertriglyceridemia, may not have confounding roles; they might however be on the causal pathway from elevated BMI to metabolic syndrome and type 2 diabetes. Therefore, the adjusted odds ratios yield a minimal estimate of the causal effect of BMI. Population attributable risks were calculated with the use of the formula (P(OR-1)χ[P(OR-1)+1]) × 100, where P is the prevalence of the overweight or obesity in Tehranian population (exposed to the risk factor) [2] and OR is the adjusted odds ratio [23]. The 95 percent confidence interval (CI) was calculated by determining the 95% CI for log(P [OR-1]) on the basis of the standard errors for P and OR and transforming back to the 95% CI for the population attributable risk. All statistical analyses were performed using SPSS software (version 13.0; SPSS Inc. Chicago, Ill, USA). All statistical tests were two sided and differences with probability values < 0.05 were considered statistically significant.

## Results

Of the 4728 subjects studied, aged 42.9 ± 13.6 years at the beginning of the study, 41.5% (n = 1961) were male and 58.5% (n = 2767) were female. Overall, 42.3% (n = 2001) were overweight and 23.7% (n = 1121) were obese. The proportion of obesity was significantly higher in women than in men (29.8% vs. 15.1%, respectively;*P *< 0.001). Furthermore, underweight subjects were more likely to be current smokers (16.7%) and obese subjects were more likely to be non-smoker (87.1%) (Linear-by-linear association χ^2 ^= 44.4, *P *< 0.001). Also, a positive family history of diabetes was reported more in obese subjects than in normal and underweight ones (*P *< 0.001). Systolic and diastolic blood pressures as well as serum triglycerides were positively related to BMI category (Table 1). Almost similar patterns were observed in men (Additional file 1) and women (Additional file 2).

**Table 1:** General characteristics and biochemical variables among the four categories of body mass index (BMI)

Variable		**BMI Category (kg/m^2^)**				
			
	**All**	**Underweight < 18.5**	**Normal 18.5 to < 25**	**Overweight 25 to < 30**	**Obese ≥ 30**	***P *for trend**
Number (%)	4728 (100)	90 (1.9)	1516 (32.1)	2001 (42.3)	1121 (23.7)	
Age (years)	42.9 ± 13.6	34.3 ± 14.7	40.0 ± 14.4	44.0 ± 13.1	45.4 ± 12.1	< 0.001
Female (%)	58.5	56.7	49.4	57.1	73.6	< 0.001
Family history of diabetes (%)	27.0	17.2	23.9	28.0	30.1	< 0.001
Current cigarette smoker (%)	11.9	16.7	15.5	11.3	7.5	< 0.001
Non-smoker (%)	81.2	78.9	77.3	81.0	87.1	< 0.001
Systolic blood pressure (mmHg)	119 ± 18	105 ± 13	114 ± 16	120 ± 18	124 ± 19	< 0.001
Diastolic blood pressure (mmHg)	78 ± 10	70 ± 8	74 ± 10	78 ± 10	82 ± 10	< 0.001
Fasting plasma glucose (mmol/L)	5.00 ± 0.54	4.74 ± 0.39	4.88 ± 0.51	5.03 ± 0.54	5.13 ± 0.56	< 0.001
2-hour postchallenge plasma glucose (mmol/L)	5.95 ± 1.64	4.94 ± 1.33	5.50 ± 1.48	6.07 ± 1.64	6.45 ± 1.68	< 0.001
Triglycerides (mmol/L)	1.88 ± 1.17	1.00 ± 0.50	1.50 ± 0.92	2.03 ± 1.25	2.21 ± 1.22	< 0.001
Total cholesterols (mmol/L)	5.44 ± 1.16	4.56 ± 0.94	5.08 ± 1.08	5.54 ± 1.14	5.80 ± 1.15	< 0.001
HDL-C (mmol/L)	1.09 ± 0.28	1.29 ± 0.31	1.13 ± 0.29	1.08 ± 0.28	1.07 ± 0.27	< 0.001
LDL-C (mmol/L)	3.49 ± 0.97	2.81 ± 0.86	3.26 ± 0.93	3.56 ± 0.96	3.72 ± 0.95	< 0.001

HDL-C, High density lipoprotein-cholesterol; LDL-C, Low density lipoprotein cholesterol

During the 3.6-year follow-up period, 3.8% of the participants (n = 182) developed diabetes, of whom, 59.9% (n = 109) were women. Of the normal, overweight, and obese subjects 1.4%, 3.6%, and 7.8% developed diabetes, respectively, while none of the 90 underweight subjects became diabetic during the follow-up period. Hence, we excluded the underweight subjects from additional analyses.

The ORs for incident diabetes were 2.57 (95% CI 1.60 to 4.13) for overweight and 5.79 (95% CI 3.63 to 9.23) for obesity when compared to normal BMI. Table 2 shows that stepwise adjustments for family history of diabetes, age, triglycerides, and systolic blood pressure attenuated the ORs of incident diabetes in overweight and obese subjects, an attenuation which was greater in the obese than in the overweight group and also, was less when adjusted for systolic blood pressure than the other variables (Table 2). The same pattern was observed in both sexes. Furthermore, the crude and adjusted ORs were higher in women than men for both overweight and obese participants. Adjusted ORs for incidence diabetes in overweight men were not statistically significant.

**Table 2:** Odds ratios for incident diabetes in 3.6-year follow-up in 4638 subjects (1922 men and 2716 women) without diabetes at baseline by the three main categories of body mass index (BMI)

		**Total (n = 4638)**			**Men (n = 1922)**			**Women (n = 2716)**	
BMI (kg/m^2^)	**18.5 to < 25**	**25 to < 30**	**≥ 30**	**18.5 to < 25**	**25 to < 30**	**≥ 30**	**18.5 to < 25**	**25 to < 30**	**≥ 30**

Number (%)	1516	2001	1121	767	859	296	749	1142	825
Number of incident diabetes (%)	23 (1.4)	72 (3.6)	87 (7.8)	15 (2.0)	32 (3.7)	26 (8.8)	8 (1.1)	40 (3.5)	61 (7.4)
Unadjusted odds ratios (95% CI)	1	2.57 (1.60–4.13)	5.79 (3.63–9.23)	1	1.9 (1.04 – 3.61)	4.83 (2.52 – 9.25)	1	3.36 (1.57–7.22)	7.39 (3.52–15.56)
Adjusted odds ratios (95% CI)									
Family history of diabetes (FHD)	1	2.48 (1.53–4.03)	5.36 (3.32–8.65)	1	1.82 (0.95 – 3.47)	4.69 (2.40–9.15)	1	3.27 (1.52–7.03)	6.57 (3.11–13.90)
FHD & age	1	2.20 (1.35–3.58)	4.65 (2.87–7.52)	1	1.67 (0.87–3.20)	4.68 (2.39–9.18)	1	2.78 (1.29–6.01)	5.17 (2.42–11.0)
FHD & age & triglycerides	1	1.89 (1.15–3.09)	3.89 (2.39–6.32)	1	1.31 (0.67–2.56)	3.82 (1.93–7.55)	1	2.54 (1.17–5.51)	4.46 (2.07–9.60)
FHD & age & triglycerides & systolic blood pressure	1	1.76 (1.07–2.89)	3.54 (2.16–5.79)	1	1.2 (0.62–2.40)	3.52 (1.76–7.04)	1	2.43 (1.12–5.29)	4.15 (1.92–8.97)

Population attributable risk (%) (95% CI)		23.3 (21.3–25.3)	37.1 (36.2–37.9)		7.8 (3.8–11.8)	26.6 (24.6–28.7)		35.3 (31.7–38.8)	48.34 (47.1–49.6)

Population attributable risk percentages comparing overweight and obesity with normal BMI (< 25 kg/m^2^) and based on odds ratios obtained from the models adjusted for family history of diabetes, age, triglycerides, and systolic blood pressure were 23.3% (95% CI 21.3% to 25.3%) and 37.1% (95% CI 36.2% to 37.9%), respectively. That is 60.4% of total incident of T2DM could be attributed to excess weight in the general population of Tehran; while, this figure was 83.6% in women but 34.4% in men.

## Discussion

This study shows that in an urban population during a short period of follow-up, there is a strong association between excess weight and risk of diabetes; despite the association being reduced by adjustment of some well known related factors, it remained considerable. In general, more than a half of the disease burden of diabetes could be attributed to excess weight. In our study, furthermore, compared with normal individuals, the adjusted odds ratios for incident type 2 DM for overweight and obese individuals were 1.76 and 3.54, respectively. Moreover, the adjusted population attributable risk of developing type 2 DM for overweight was 23.3% and for obesity was 37.1%. Noticeably, while in women, 83.6% of new cases of diabetes could be attributed to excess weight, in men, almost one third of incident diabetes was attributed to overweight or obesity.

These findings are in accordance with the results of previous studies, which have consistently demonstrated the association between BMI and increased risk of diabetes [8-11,15]. These studies differ in terms of age and sex of their study population, length of follow-up time, and the variables selected for adjustment. Studies with short follow-up, similar to ours, have reported strong relationships between excess weight and the risk of developing diabetes. During a 5-year follow-up in a cohort of 51529 male, health professionals, 40–75 years of age, those with a BMI of 35 and over had a multivariate adjusted relative risk of 42.1 (CI 95% 22 to 86) compared with men who had a BMI < 23 [8]. In our study with shorter follow-up time (i.e. 3.6 years) and after adjustment for age, family history of diabetes, triglycerides and systolic blood pressure, obese individuals with average age of 45.4 years, had a 3.5 times greater risk than those who had BMI < 25 kg/m^2 ^at baseline. Other studies with longer follow-ups have also demonstrated this association. A study of Scottish men and women, aged 45–64 years, with follow-ups of over 28 years, reported an adjusted odds ratio equal to 18.5 for an obese group in comparison to a normal weight group [17]. The fat cell can be considered as a type of endocrine cell, and adipose tissue an endocrine organ [24]. Adipocytes secrete a number of adipocyte hormones and cytokines. These adipocyte hormones and adipokines therefore may increase the risk of diabetes via several pathways such as increasing insulin resistance [24]. In addition, several studies have shown that in high-risk populations, weight loss can reduce the risk of diabetes. The Diabetes Prevention Program Research Group study found that lifestyle interventions implemented over 2.8 years reduced the incidence of diabetes by 58% [25]. Likewise, a modest weight loss of one kg was associated with a 33% lower risk of diabetes [16].

Although, the association between BMI and increased risk of diabetes was significant in the population studied, this association was stronger in women than men; the crude and adjusted ORs being almost 1.5 to 2 times higher in women than men (table 2). This can be explained by the fact that obesity was more prevalent in women. Moreover, lower levels of physical activity in women than men, weight gains in pregnancy and not returning to the optimal weight, may be other causes of the higher rate of obesity and incident diabetes in women [2]. This interpretation is in accordance with previous findings from the Health Professional Follow-Up Study [8], and the Nurses Health Study [9] which showed that age-adjusted relative risk for incident diabetes was higher in women than men across the spectrum of BMI. Despite these explanations, however, incidence of DM is almost equal between sexes (3.7% in men vs. 3.9% in women). There are some explanations for this finding; first, absolute weight gain and increase in waist circumference, during the 3.6 years follow-up period, in men were greater than women. These changes among men may describe why the incidence of diabetes in men was almost similar to women despite higher proportions of obesity in women. Second, men were somewhat older than women were. Last but not least, the lost-to-follow-up rate was higher in women than in men. That is, some of the women who developed diabetes had been lost (data not shown).

Despite the strong association between excess weight and developing diabetes, only a few studies have reported estimates of the risk of diabetes attributable to overweight and obesity [17,24]. In the study of a Scottish population, population attributable risk percentages comparing those who were in the high normal weight group or above, to those in the low normal weight group (18.5 to < 22.5 kg/m2) were around 60% [17]. The estimated population attributable risk of the effect of obesity on incident diabetes was considerable in our study, showing that thirty percent of the disease burden of diabetes in our population was due to obesity. Since diabetes is indeed a preventable disease, the causal relationship of the obesity with diabetes is well established and it is conceivable that obesity can be eliminated, the population attributable fraction is an appropriate measure to be used by health policy officials for future strategies.

Regarding both the strengths and limitations of our study, the main strength of our study is a large sample sized population-based prospective study; in this cohort, we clearly showed an independent role for overweight and obesity in the development of diabetes. In addition, given the population-based design of our study, it enabled the impact of overweight and obesity to be estimated in terms of their population attributable risk, which can help policymakers to implement appropriate programs to prevent diabetes. The several limitations of our analysis also deserve comment. First, we could not adjust some confounding variables such as physical activity, socio-economic status, and dietary habit. Second, since the number of cases of incident diabetes in subjects with normal BMI was relatively low, we were unable to show possible trends across normal BMI category. Third, we have used weight and height measured at a particular point in time; it is possible that some participants who were underweight or had normal weight at the baseline assessment will have increased weight in the intervening period or vice versa. The results of this would be to dilute any effect of the true association. Last but not least, the main limitation, a considerable fraction of subjects were lost during the follow-up period; it is possible that some participants who developed incident diabetes were among those lost, which might influence our estimates. Furthermore, because those who lost to follow-up were relatively healthier, we may overestimate the population attributable risk.

Our estimate is quite less than that of other long-term studies [17,24,26] which estimated population attributable risk for diabetes associated with excess weight. This estimate may be less if we could adjust for further risk factors of diabetes including physical activity, socio-economic status, and dietary habit. This implies that in short-term intervals, contribution of other risk factors such as smoking, physical activity, poor diet, and weight change may be more important compared to long-term follow-up [26]; this may be noticeable particularly in men. Accordingly, one may speculate that in order to implement short-term preventive strategies, paying more attention to other risk factors of diabetes might be reasonable.

## Conclusion

In summary, our results indicate that incident type 2 DM is largely attributable to excess weight. In other words, our quantification shows that over 60% disease burden of diabetes in our population is attributable to overweight and obesity. This pattern is strongest in women as compared to men. Therefore, prevention of excess weight needs to be prioritized as a major strategy for reducing incidence of type 2 DM in our population.

## Abbreviations

2h-PG: 2-hour postchallenge Plasma Glucose

ANOVA: Analysis Of Variance

BMI: Body Mass Index

CVD: Cardiovascular Disease

DBP: Diastolic Blood Pressure

FPG: Fasting Plasma Glucose

HDL-C: High Density Lipoprotein – Cholesterol

LDL-C: Low Density Lipoprotein – Cholesterol

OGTT: Oral Glucose Tolerance Test

PAR: Population Attributable Risk

SBP: Systolic Blood Pressure

SD: Standard Deviation

T2DM: Type 2 Diabetes Mellitus

TC: Total Cholesterol

TG: Triglycerides

TLGS: Tehran Lipid and Glucose Study

## Competing interests

The author(s) declare that they have no competing interests.

## Authors' contributions

• **F.H. **contributed to design of the study, manuscript preparation, and reading and approval of the final manuscript.

• **M.R. **contributed to design of the study, analysis of data, and writing of the manuscript and its revisions, and reading and approval of the final manuscript.

• **F.A. **contributed to provision of data, and reading and approval of the final manuscript.

## Pre-publication history

The pre-publication history for this paper can be accessed here:



## Supplementary Material

Additional file 1 General characteristics and biochemical variables among the four categories of body mass index (BMI) in 1961 male participants. The data provided  represent increasing prevalence of traditional cardiovascular risk factors (except for HDL) by increasing body mass index in men. Click here for file

Additional file 2 General characteristics and biochemical variables among the four categories of body mass index (BMI) in 2767 female participants. The data provided  represent increasing prevalence of traditional cardiovascular risk factors (except for smoking) by increasing body mass index in women. Click here for file
