# Supplementary material for: Population attributable risk for diabetes associated with excess weight in Tehranian adults: a population-based cohort study
Source: BMC Public Health. 2007 Nov 14;7:328. doi: 10.1186/1471-2458-7-328 (PMC2204006; doi:10.1186/1471-2458-7-328)
Supplement: Additional file 1 — General characteristics and biochemical variables among the four categories of body mass index (BMI) in 1961 male participants. The data provided represent increasing prevalence of traditional cardiovascular risk factors (except for HDL) by increasing body mass index in men. [file 1471-2458-7-328-S1.doc]

General characteristics and biochemical variables among the four categories of body mass index (BMI) in 1961 male participants

| Variable | **Men** | **BMI Category (kg/m^2^)** | | | | ***P* for trend** |
| --- | --- | --- | --- | --- | --- | --- |
|  |  | **Underweight**  **<18.5** | **Normal**  **18.5 to <25** | **Overweight**  **25 to <30** | **Obese**  **≥30** |  |
| Percent | 100 | 2.0 | 39.1 | 43.8 | 15.1 |  |
| Age (years) | 44.7±14.4 | 41.5±16.6 | 43.5±15.4 | 46.0±13.6 | 44.6±13.6 | <0.001 |
| Family history of diabetes (%) | 25.4 | 13.5 | 22.2 | 26.4 | 32.2 | <0.001 |
| Current cigarette smoker (%) | 21.8 | 38.5 | 25.6 | 19.3 | 17.2 | <0.001 |
| Non-smoker (%) | 75.5 | 61.5 | 72.5 | 77.4 | 79.7 | <0.001 |
| Systolic blood pressure (mmHg) | 120.6±17.4 | 109±16.3 | 116.7±16 | 122.9±17.8 | 125.6±17.1 | <0.001 |
| Diastolic blood pressure (mmHg) | 77.9±10.6 | 71.2±8.1 | 74.5±10.1 | 79.5±10.2 | 82.6±10.5 | <0.001 |
| Fasting plasma glucose (mmol/L) | 5±0.5 | 4.8±0.4 | 4.9±0.5 | 5.1±0.5 | 5.2±0.5 | <0.001 |
| 2-hour postchallenge plasma glucose (mmol/L) | 5.7±1.7 | 4.8±1.4 | 5.3±1.6 | 5.9±1.7 | 6.2±1.8 | <0.001 |
| Triglycerides (mmol/L) | 2±1.3 | 1.1±0.6 | 1.7±1.0 | 2.3±1.4 | 2.4±1.2 | <0.001 |
| Total cholesterols (mmol/L) | 5.3±1.1 | 4.4±0.9 | 5.1±1.1 | 5.5±1.0 | 5.6±1.0 | <0.01 |
| HDL-C (mmol/L) | 0.1±0.2 | 1.2±0.2 | 1.0±0.2 | 0.9±0.2 | 0.9±0.2 | 0.082 |
| LDL-C (mmol/L) | 3.4±0.9 | 2.8±0.7 | 3.3±0.9 | 3.5±0.9 | 3.6±0.9 | <0.01 |

HDL-C, High density lipoprotein-cholesterol; LDL-C, Low density lipoprotein cholesterol
